# Overview of national strategies for the prevention and management of non-communicable diseases

**DOI:** 10.1093/eurpub/ckac130.047

**Published:** 2022-10-25

**Authors:** I Reinsperger, L Gassner, I Zechmeister-Koss

**Affiliations:** Austrian Institute for HTA, Vienna, Austria

## Abstract

**Background:**

Several countries have developed national strategies or policies for preventing and managing non-communicable diseases (NCDs) which are the leading cause of death worldwide. We aim to provide an overview of these strategies from selected countries and their implementation, focusing on chronic respiratory and cardiovascular diseases, diabetes and depression.

**Methods:**

Using a comprehensive structured hand search, strategies from 8 countries (Germany, Switzerland, Netherlands, Finland, Ireland, United Kingdom, Canada, Australia) were identified and information on the main characteristics and implementation process of the strategies was extracted.

**Results:**

A total of 18 strategies were included. Most of the strategies formulate rather broad overarching aims or visions (e.g., “stay healthy” or “living healthier lives”) as well as more specific targets that differ across strategies, e.g. focusing on improving quality of life and health literacy, reducing health inequalities or strengthening integrated care. The level of detail of information on implementation, monitoring and evaluation processes as well as financing is very heterogeneous. All strategies provide information on activities to achieve their aims, e.g. in the areas of health promotion/primary prevention, self-management, screening, integrated care, measures for specific risk groups or activities outside the health sector. Only a few strategies mention specific, already implemented (and evaluated) interventions, such as prevention or disease management programmes.

**Conclusions:**

The included NCD strategies differ considerably in terms of level of detail, structure and implementation. We focused on interventions within the health sector and on adults as a target group. However, for the prevention and management of NCDs, it is important to start in early childhood and to adequately address the social determinants of health with a ‘Health in All Policies’ approach.

**Key messages:**

- Many countries have developed and implemented strategies to strengthen and coordinate action to tackle NCDs which are a growing problem worldwide due to their high mortality and morbidity.
- A structured approach from the macro to the micro level seems crucial for a comprehensive, coordinated overall policy for preventing and managing NCDs.

